# Editorial: Language across neurodevelopmental disorders

**DOI:** 10.3389/fpsyg.2022.1121997

**Published:** 2023-01-04

**Authors:** Marisa G. Filipe, Lénia Carvalhais, Leonard Abbeduto, Sónia Frota

**Affiliations:** ^1^Center of Linguistics, School of Arts and Humanities, University of Lisbon, Lisbon, Portugal; ^2^Infante D. Henrique Portucalense University, Porto, Portugal; ^3^MIND Institute & Department of Psychiatry and Behavioral Sciences, University of California, Davis, Sacramento, CA, United States

**Keywords:** language, development, impairments, neurodevelopment, neurodevelopmental disorders

The development of language is critical to meet the demands and challenges of contemporary societies. Unfortunately, many children do not master language skills at rates or levels consistent with their chronological ages, and language impairments during childhood tend to persist across the development with lifelong implications for academic, social-emotional, and behavioral functioning (Conti-Ramsden et al., 2018). Although many children diagnosed with neurodevelopmental conditions such as autism and intellectual disability display language impairments, the specific profile of impairments may differ across disorders, making research investigating language across these clinical conditions critical. Neurodevelopmental disorders imply disruptions in the typical growth and development of the central nervous system (e.g., Goldstein and Reynolds, 1999), and the symptoms usually emerge early in childhood (Bishop and Rutter, 2008).

The set of papers gathered on the present Research Topic provides evidence on the relationship between prelinguistic communication, language (oral and written), and several neurodevelopmental conditions, namely autism, fragile X syndrome, Down syndrome, and Developmental Language Disorder. The papers include a multidisciplinary list of contributors with different disciplinary backgrounds (e.g., psychologists, speech and language therapists, linguists, and practitioners), each one contributing in a unique way to our knowledge about language in one or more neurodevelopmental disorders. This collection included original research papers (cross-sectional and longitudinal designs), perspective articles, and reviews dealing with both theoretical and practical issues.

In a perspective article, Weismer and Saffran present a fruitful and interesting line of research. The authors argue that individual differences in statistical learning (i.e., the detection of patterns with stable probabilities) among children with autism, together with the prediction deficits (hyperplasticity) characteristic of autism, may be related to the variability in the structural language in children diagnosed with this condition.

Ravi et al. report on a study in which they found that infants with better social communication skills at 12 months present better language scores at 24 months. However, infants who met criteria for autism did not show this developmental coupling until 24 months of age. The authors suggest that social communication outcomes shape downstream language skills and highlight the need to support the development of social communication skills prior to a formal autism diagnosis.

Mankovich et al., through a recurrence quantification analysis (i.e., a technique to understand how units of speech repeat across stretches of transcriptions), found that grammatical productivity and lexical productivity were related to language competence in different ways in a sample of children with autism. These findings indicated that beyond traditional linguistic analysis, recurrence analysis may be helpful to reveal differences in the spoken language of individuals with autism.

Zheng et al. explored how the measurement of autism symptoms might be affected by language and developmental levels. Even for children with minimal verbal abilities, the authors highlight the need for finer distinctions based on spoken language level and/or mental age to optimize the measurement of autism symptoms.

Reetzke et al. emphasize the need for effective community-based implementation strategies for young autistic children from low-resourced households. In particular, these authors found that young children with autism from the lowest-resource households exhibited the poorest language and social communication skills, as well as the poorest non-verbal problem-solving and fine-motor abilities, along with more features of attention-deficit/hyperactivity disorder and atypical auditory processing.

Venker and Johnson explored the relationship between the use of electronic toys and the quantity and lexical diversity of spoken language produced by children with autism and neurotypical children matched on chronological age (2–5 years). They found that children with autism and their neurotypical peers talked significantly less and produced significantly fewer unique words during electronic toy play compared to traditional toy play. The authors suggest that play-based interventions for children with autism may be most effective when they incorporate traditional toys rather than electronic toys.

Moving from spoken language to reading abilities, Vale et al., in a systematic literature review, showed that the majority of children with autism have well preserved word reading abilities. However, word reading strategies in those with autism are far from being completely understood. The authors emphasize that there is much that remains unknown about the specific word reading difficulties and strengths of children with autism.

The set of papers below focused on other neurodevelopmental conditions beyond autism. Thurman and Nunnally found join attention differences between preschool boys with autism or fragile X syndrome when controlling for the influence of age, non-verbal IQ, and autism symptom severity. In addition, differences between the two groups of children were also observed when considering how joint attention performance related to other aspects of the phenotype. Taken together, these findings have implications for understanding phenotypic differences in the development of joint attention, as well as treatments, for these two conditions.

In a systematic review, Hoffmann explored language patterns of weakness and strengths for individuals with fragile X syndrome, highlining the specific role of cognition, autistic symptomatology, and gender. Importantly, this paper presents implications for assessment and intervention practices.

Filipe et al. explored the evidence for early predictors of language outcomes in infants and toddlers with Down Syndrome. Results indicated that child-related factors such as maternal educational level and parents' translation of their children's gestures into words predict language outcomes in Down syndrome. In addition, the level of adaptive functioning, cognitive function, attention skills, communicative intent, early vocalizations, gestures, baby signs, and vocabulary level of the child are also significant predictors of language outcomes in this population.

Prahl and Schuele explored the reliability and validity of several commonly used measures of listening and reading comprehension in terms of their utility for individuals with Down syndrome. Overall, the authors found strong evidence of reliability and construct validity for three of four measurement methods; namely, non-verbal response, cloze procedure, and passage-level with open-ended questions.

Angulo-Chavira et al. presented clues about how people with Down process and extract information from speech and in context. The authors examined whether young adults with Down syndrome anticipate a referent in the same way as their typical development peers matched by mental age and gender. It was found that participants with Down syndrome predicted nouns in closely related verb-noun pairs but not in pairs that were moderately related and in which they needed visual context to generate the prediction. These processing differences may provide insights into therapeutic targets.

Soares et al. aimed to provide new insights into the role that explicit learning mechanisms play in the implicit learning deficits in preschool children with developmental language disorders by collecting behavioral and neuropsychological data. The findings failed to support the compensatory role of explicit learning mechanisms in the implicit learning impairments characteristic of these children.

Loveall et al. conducted a systematic review and found that the representation of individuals with neurodevelopmental disorders in the normative samples of norm-referenced language assessment tools is very low. The authors argue that test developers should (i) include these individuals as part of the iterative test development, (ii) assess a high number of individuals with disabilities in the normative samples, and (iii) include separate norms for individuals with disabilities. The failure to do so limits the usefulness of these tests for both research and clinical purposes.

Overall, the papers in this Research Topic should be of interest to researchers, teachers, educators, clinicians, and students interested in understanding language across neurodevelopmental disorders. Although the nature and extent of the connections between language and neurodevelopmental disorders continue to be investigated, it is now clear that specific language impairment profiles can be identified, and at different levels, across neurodevelopmental disorders. The papers in this Research Topic reflect recent advances in the field, providing some insight into approaches to inform educational and clinical services related to language intervention. We hope these papers will motivate further research and move the field forward in ways that lead to better functional outcomes for affected children and youth.

## Author contributions

MF prepared the first draft of the manuscript. All authors contributed to the article and approved the submitted version.
